# Supplementary material for: The practice of reaction window in an electrocatalytic on-chip microcell
Source: Nat Commun. 2023 Oct 27;14:6838. doi: 10.1038/s41467-023-42645-0 (PMC10611802; doi:10.1038/s41467-023-42645-0)
Supplement: Supplementary file 2 — Supplementary Information [file 41467_2023_42645_MOESM2_ESM.pdf]

Supplementary Information

The practice of reaction window in an electrocatalytic on-chip microcell

Hang Xia[1], Xiaoru Sang[1], Zhiwen Shu[2], Zude Shi[1], Zefen Li[3], Shasha Guo[4], Xiuyun An[1], Caitian Gao[5,6*], Fucai Liu[3], Huigao Duan[2,6], Zheng Liu[4*], and Yongmin He[1,6*]

[1]State Key Laboratory of Chemo/Biosensing and Chemometrics, College of Chemistry and Chemical Engineering, Hunan University, Changsha 410082, P. R. China.
[2]College of Mechanical and Vehicle Engineering, National Engineering Research Centre for High Efficiency Grinding, Hunan University, Changsha 410082, P. R. China.
[3]School of Optoelectronic Science and Engineering, University of Electronic Science and Technology of China, Chengdu 610054, P. R. China.
[4]School of Materials Science and Engineering, Nanyang Technological University, Singapore 639798, Singapore.
[5]School of Physics and Electronics, Hunan University, Changsha 410082, P. R. China.
[6]Greater Bay Area Institute for Innovation, Hunan University, Guangzhou 511300, P. R. China.

*Email: ymhe@hnu.edu.cn, z.liu@ntu.edu.sg, ctgao@hnu.edu.cn

Contents

Supplementary Figure 1. Characterization of CVD-grown MoS_2 monolayer.
Supplementary Figure 2. The four-electrode microcell for *in-situ* electronic/electrochemical testing.
Supplementary Figure 3. Configuration of the four-electrode microcell.
Supplementary Figure 4. The morphological and structural characterization during the multistep dissolution procedures.
Supplementary Figure 5. Irreversible damage of MoS_2 materials under excessive electron beam current or dose.
Supplementary Figure 6. Calibration of the microcell based on the Pt microelectrode.
Supplementary Figure 7. Transfer characteristics of monolayer MoS_2.
Supplementary Figure 8. The zoom-in HER polarization curves in Figure 2c.
Supplementary Figure 9. Full Tafel plots of MoS_2 microcells in this work.
Supplementary Figure 10. Raman spectrum of WSe_2 and $WSe_{1.8}Te_{0.2}$ nanosheets.
Supplementary Figure 11. Transfer characteristics of $WSe_{1.8}Te_{0.2}$ nanosheet.
Supplementary Figure 12. Raman spectrum of the mechanical exfoliated $NbSe_2$.
Supplementary Figure 13. Raman spectrum of the mechanical exfoliated $PtTe_2$.
Supplementary Figure 14. Transfer characteristics of $NbSe_2$ and $PtTe_2$ nanosheets.
Supplementary Figure 15. Large cell-to-cell variation in MoS_2 in-plane microcell with full-open windows.
Supplementary Figure 16. Fabrication procedure of the MoS_2/graphene heterostructure microcell.
Supplementary Figure 17. Typical polarization curves of three kinds of monolayer MoS_2 on-chip microcells.
Supplementary Figure 18. Stability of monolayer MoS_2 in full-open and vertical microcells.
Supplementary Figure 19. Vertical charge transport for $WSe_{1.8}Te_{0.2}$ nanosheet in oxygen evolution reaction.
Supplementary Figure 20. AFM images of $WSe_{1.8}Te_{0.2}$ nanosheets in microcells.
Supplementary Figure 21. Transfer curves and subthreshold slope (*SS*) values of monolayer MoS_2 microcells with the full-open and half-open windows.
Supplementary Figure 22. The strong modulation ability under EDL-gating in the on-chip microcell.
Supplementary Note 1. The calculation processes of *in-situ* resistivity of 2D catalysts.
Supplementary Note 2. The discussion about the threshold between good and bad conductance.
Supplementary Note 3. The penetration depth of surface conductance under EDL-gating.

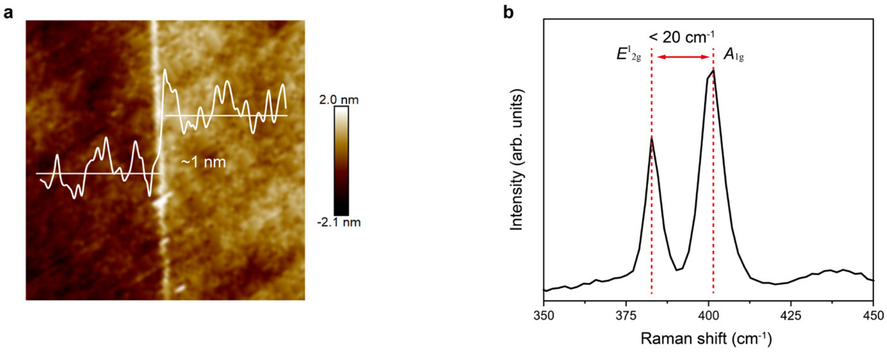

Supplementary Figure 1. Characterization of CVD-grown MoS_2 monolayer. a, The atom force microscope (AFM) image. **b,** Raman spectrum of MoS_2 monolayer.

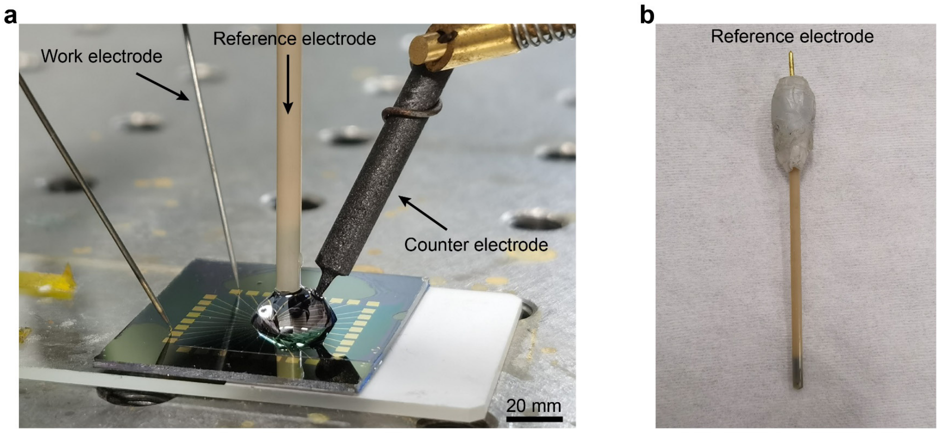

Supplementary Figure 2. The four-electrode microcell for *in-situ* electronic/electrochemical testing. a, The photograph of a four-electrode microcell. The carbon rod was polished with fine-grit sandpaper for about 10 minutes before being used as the counter electrode (CE). One tungsten needle acted as the wire connected to the working electrode (WE), and the other was used to apply the drain-source voltage (V_{ds}). **b,** The photograph of the leakless Ag/AgCl microelectrode. In all electrochemical measurements, the leakless Ag/AgCl microelectrode was used as the reference electrode (RE).

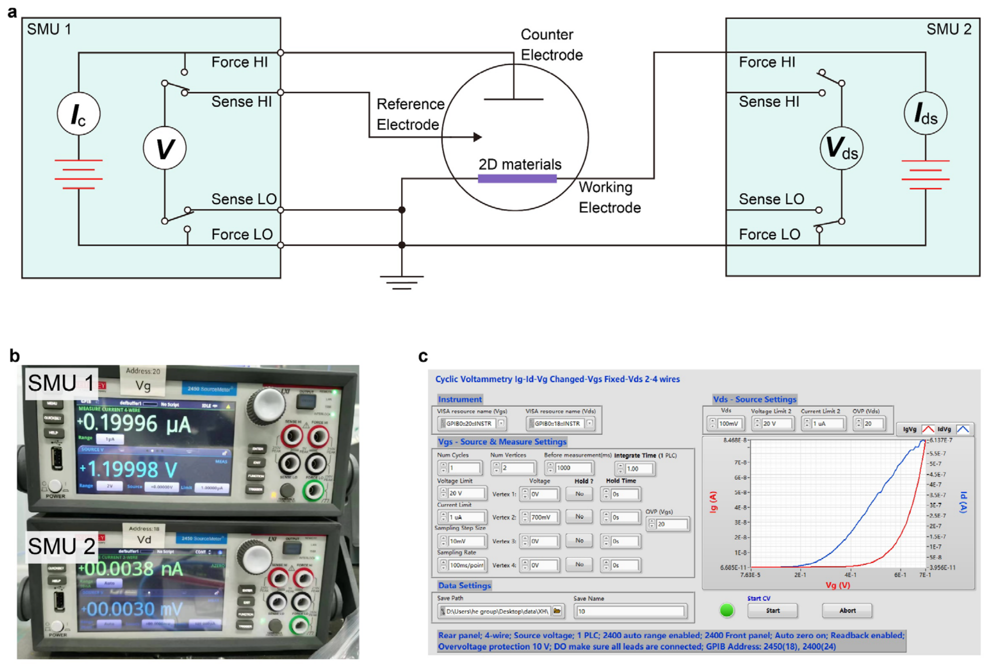

Supplementary Figure 3. Configuration of the four-electrode microcell. a, The circuit diagram of the *in-situ* electronic/electrochemical measurement, consisting of two source measurement units (two Keithley 2450, SMU 1, and SMU 2). The SMU 1 acts as the three-electrodes measurement unit. The High Force (Force HI) and High Sense (Sense HI) are connected to the CE and RE respectively. Meanwhile, the Low Sense (Sense LO) and Low Force (Force LO) both connect to the WE and the ground. The SMU 2 is only used to obtain the conductance current (I_{ds}), so the Sense HI and Sense LO are not loaded. The Force HI and Force LO in SMU 2 connect to the other end of the WE and the ground, forming a loop and collecting the electrical signals of the 2D materials. The two measurements are combined under efficient synchronous operation through the GPIB and Labview programs. Therefore, the electrochemical and electrical signals based on the 2D materials microcells can be collected simultaneously. **b,** The photograph of SMUs, which functions as described above. **c,** The interface of the home-build measurement software is based on the Labview program. The red and blue line represents the electrochemical and conductance current, respectively.

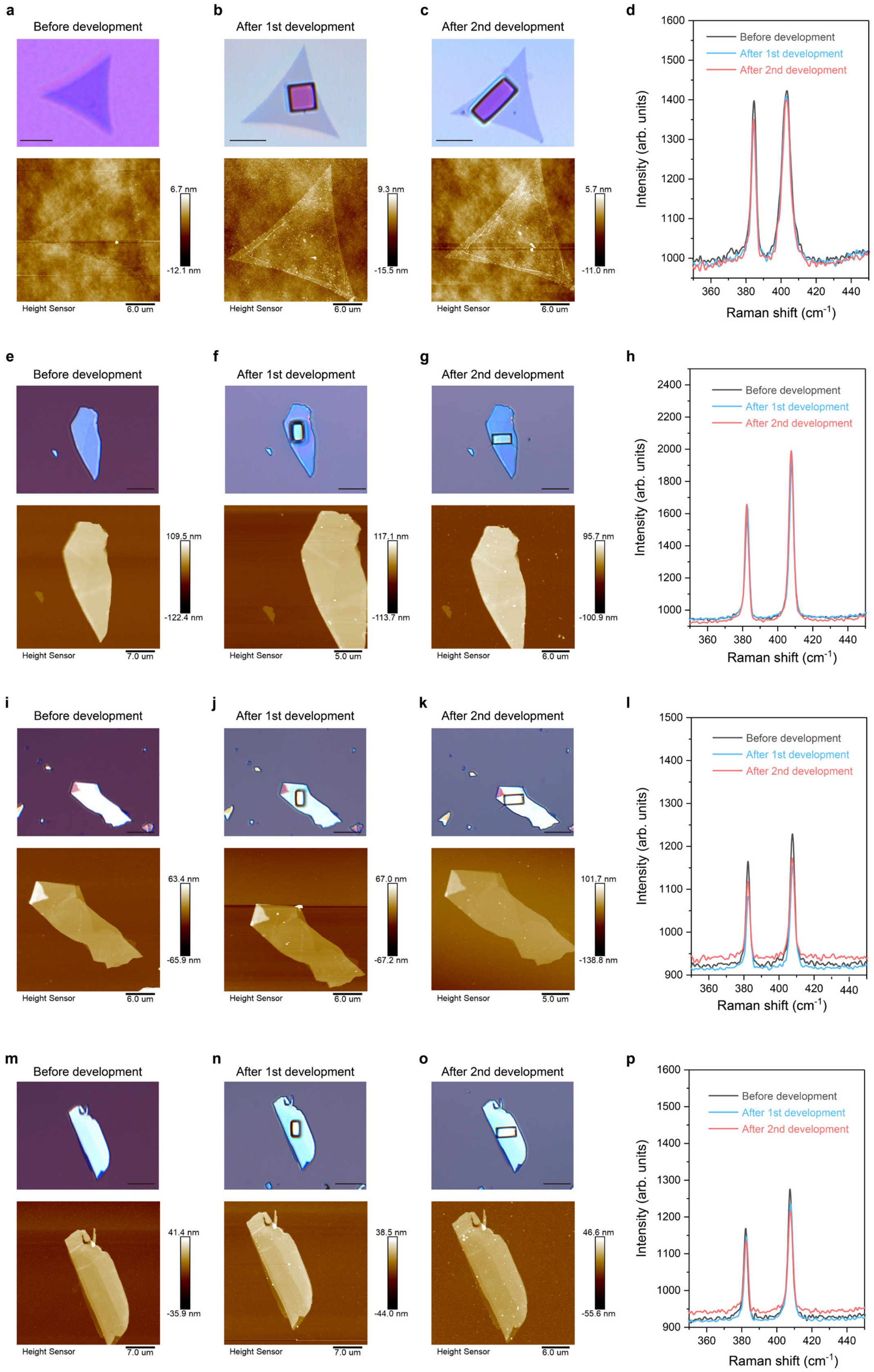

Supplementary Figure 4. The morphological and structural characterization during the multistep dissolution procedures. a-d, The optical image, AFM image, and Raman signals of monolayer MoS_2 grown by CVD before and after the development. **e-p,** The optical image,

AFM image, and Raman signals of multilayer MoS_2 nanosheets by mechanical exfoliation before and after the development. During the whole process, the properties of MoS_2 nanosheets did not change obviously. Scale bar of optical images: 10 μm.

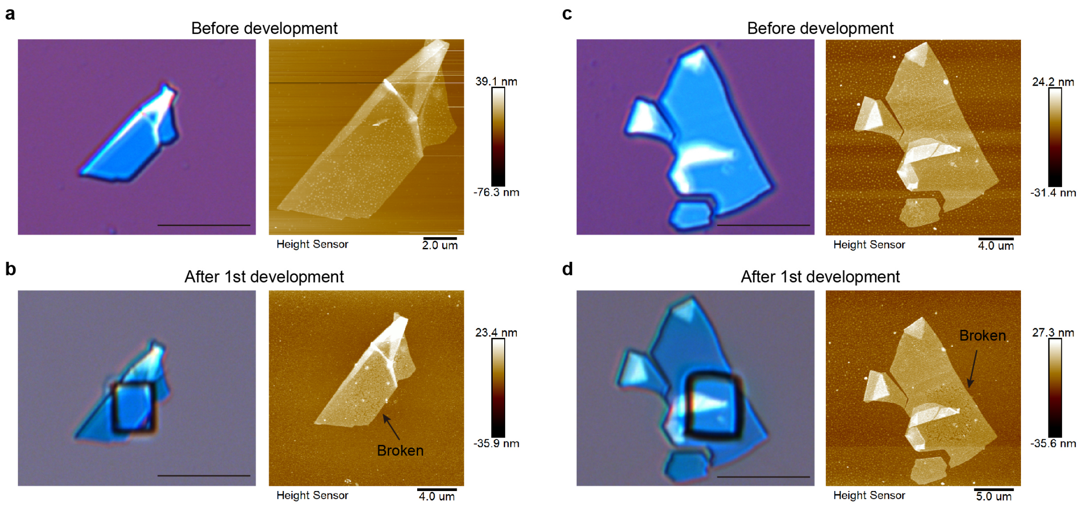

Supplementary Figure 5. Irreversible damage of MoS_2 materials under excessive electron beam current or dose. a-c, the original optical and AFM image of MoS_2 nanosheets. **b-d,** the optical and AFM image of MoS_2 nanosheet after electron beam lithography (EBL) using excessive electron beam current or dose. In our experiment, excess electron beam current (electron beam current = 690 pA in this experiment) will cause irreversible damage to the 2D materials (Supplementary Figure 5), showing small holes in the MoS_2 layer at the window regions. Scale bar of optical images: 10 μm.

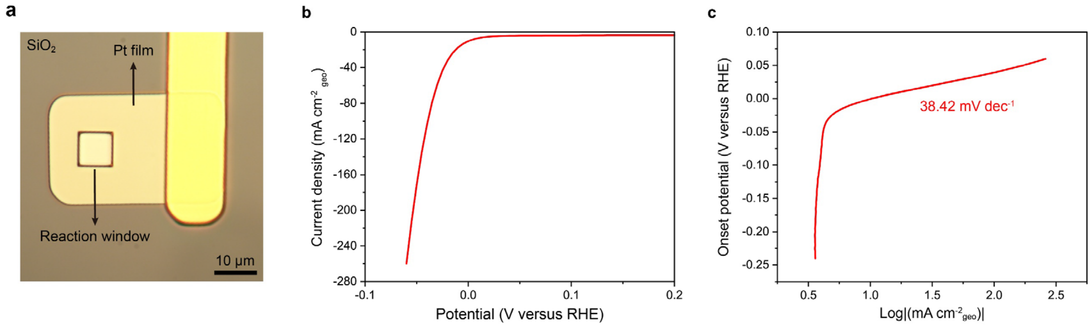

Supplementary Figure 6. Calibration of the microcell based on the Pt microelectrode. a, Optical image of Pt microelectrode with a thickness of 20 nm. **b and c,** Polarization curves of Pt microelectrode (**b**) and the corresponding Tafel slope (**c**). The results indicated the reliability of the microcell.

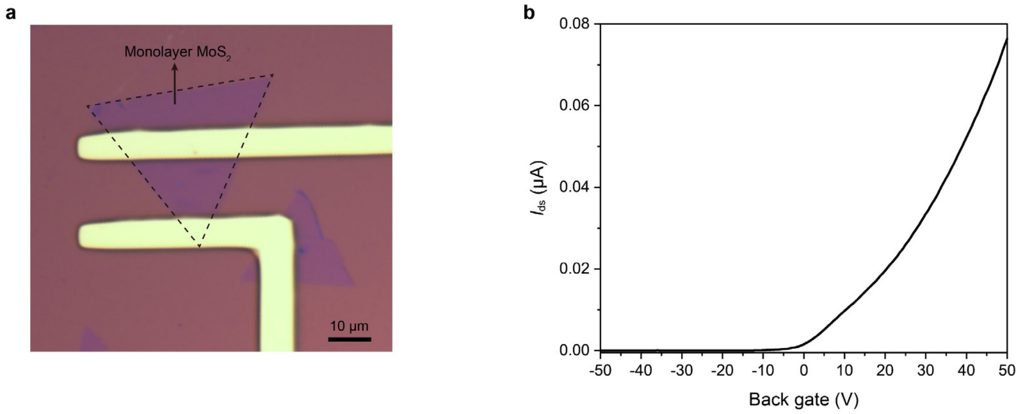

Supplementary Figure 7. Transfer characteristics of monolayer MoS_2. a, The optical photograph of the device. **b,** Back-gate measurement of the monolayer MoS_2, showing a n-type semiconducting characteristic. V_{ds} = 10 mV.

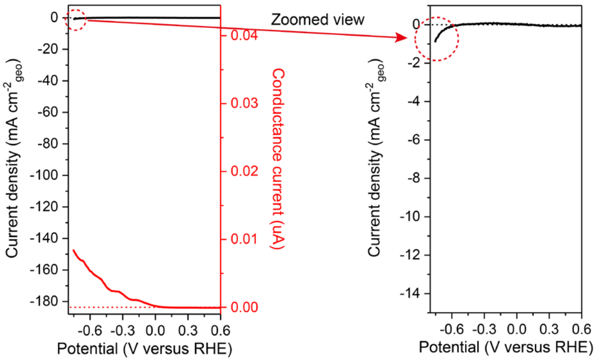

Supplementary Figure 8. The zoom-in HER polarization curves in Figure 2c.

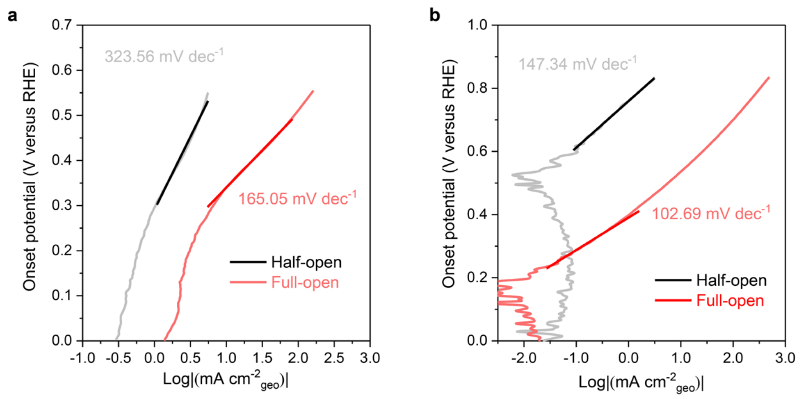

Supplementary Figure 9. Full Tafel plots of MoS_2 microcells in this work. a, The full Tafel plots of monolayer MoS_2 grown by CVD. **b,** The full Tafel plots of multilayer MoS_2 obtained by mechanical exfoliation.

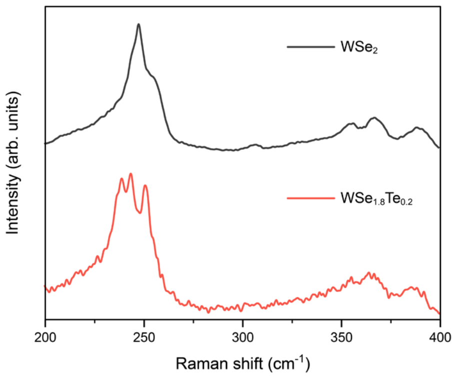

Supplementary Figure 10. Raman spectrum of WSe_2 and $WSe_{1.8}Te_{0.2}$ nanosheets.

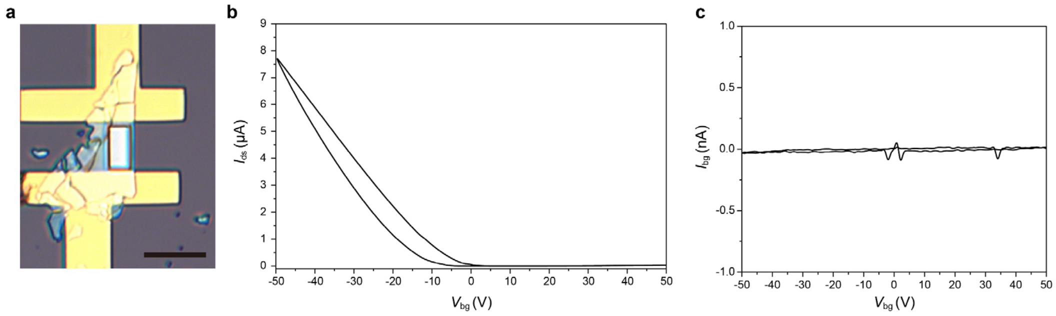

Supplementary Figure 11. Transfer characteristics of $WSe_{1.8}Te_{0.2}$ nanosheet. a, The optical image photograph of the field effect transistor device. Scale bar: 10 μm. **b,** Back-gate measurement of the $WSe_{1.8}Te_{0.2}$ nanosheet, showing strong *p*-type semiconducting characteristics. V_{ds} = 100 mV. **c,** the Leakage current of the device during the back-gate measurement.

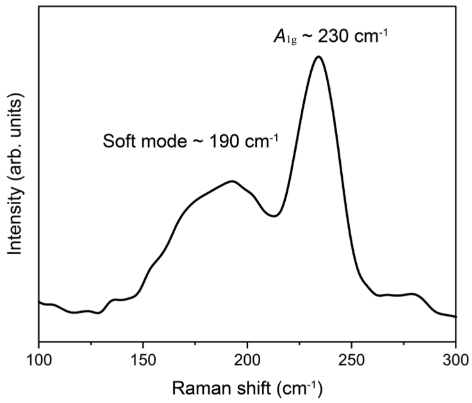

Supplementary Figure 12. Raman spectrum of the mechanical exfoliated $NbSe_2$.

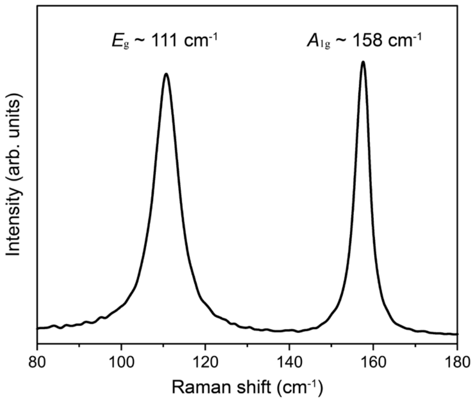

Supplementary Figure 13. Raman spectrum of the mechanical exfoliated $PtTe_2$.

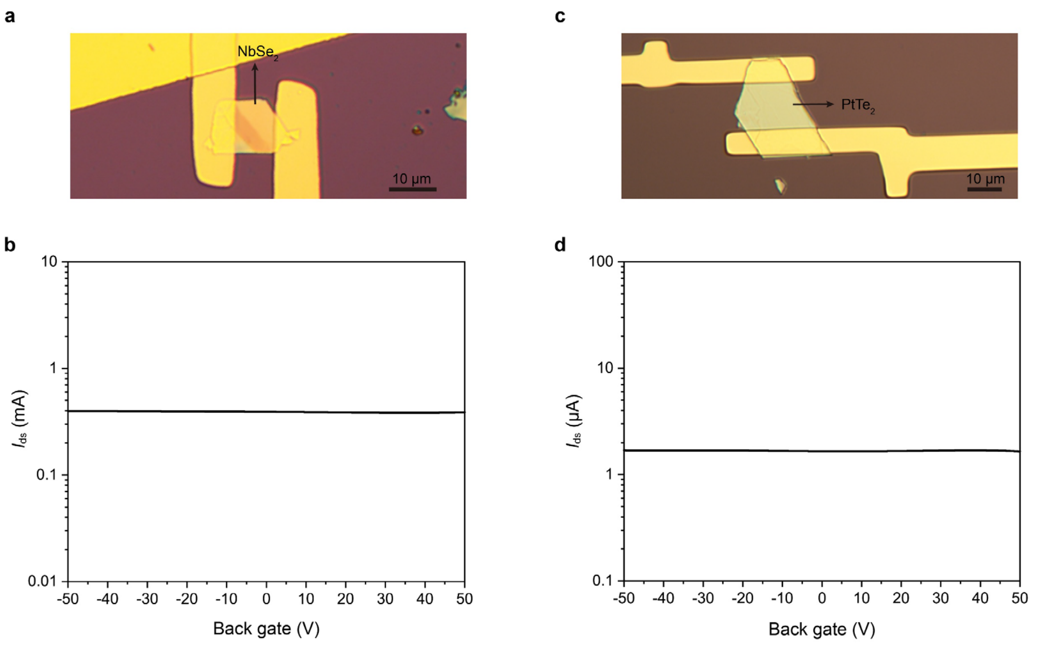

Supplementary Figure 14. Transfer characteristics of $NbSe_2$ and $PtTe_2$ nanosheets. (a-b) The optical images of $NbSe_2$ device and its corresponding back-gate measurement. V_{ds} = 100 mV. (c-d) The optical images of $PtTe_2$ device and its corresponding back-gate measurement. V_{ds} = 1 mV. Those results show the metallic behavior of $NbSe_2$ and $PtTe_2$ nanosheets.

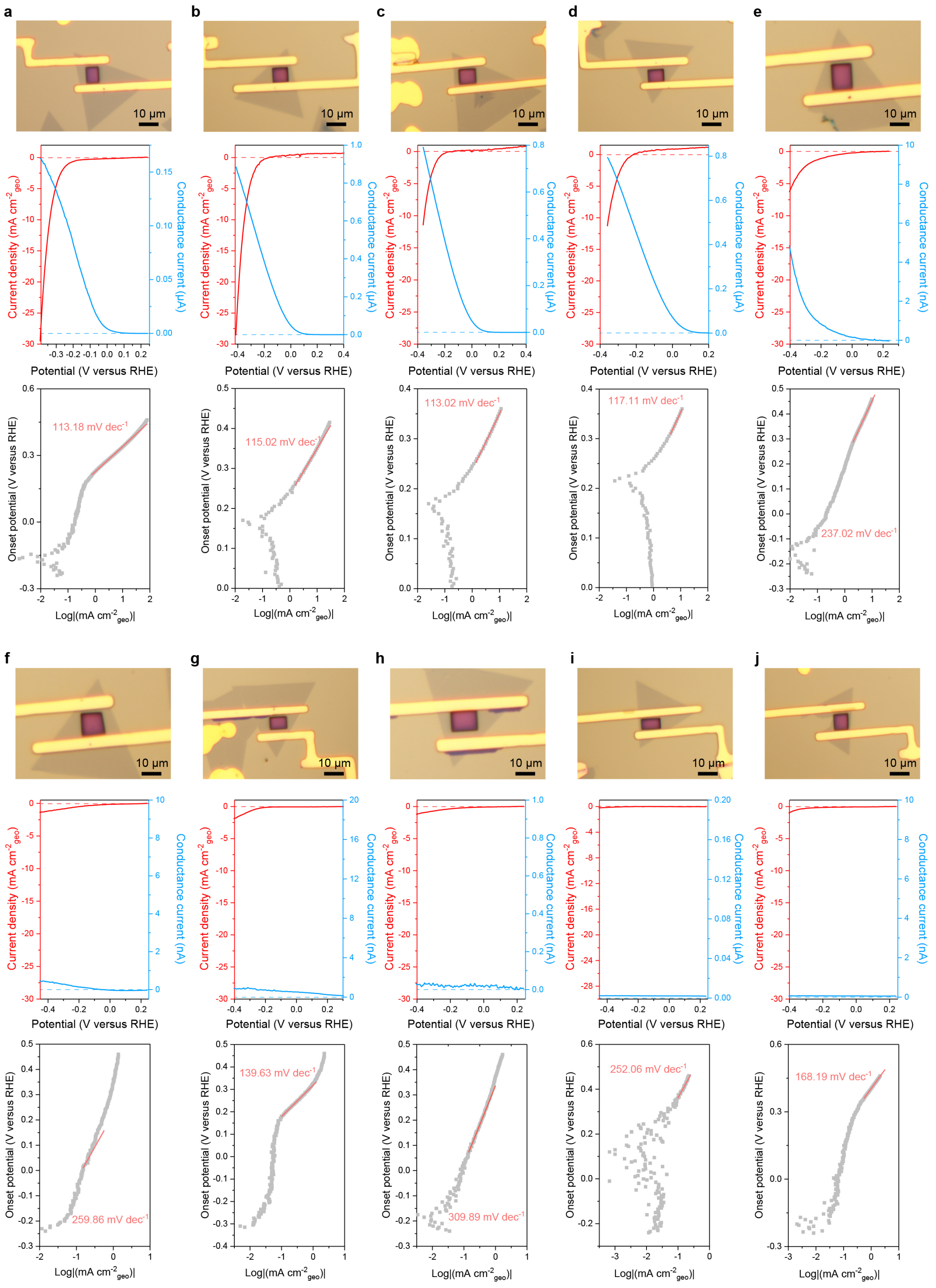

Supplementary Figure 15. Large cell-to-cell variation in MoS_2 in-plane microcell with full-open windows. Those observations demonstrate that even though all in-plane MoS_2 microcells had full-open windows, a large cell-to-cell variation was also found during measurements. Such a phenomenon would be attributed to the fluctuation of the contact barrier, a common issue in semiconductor electronic fields. This contact barrier cannot be well-tuned by EDL in the micro-cell due to the covering of the insulating layer, see Figures S15f-j. On the other hand, it is important to note that once the conductance of microcells was tuned to be

highly conductive, they can give remarkable HER properties (see Figures S15a-d).

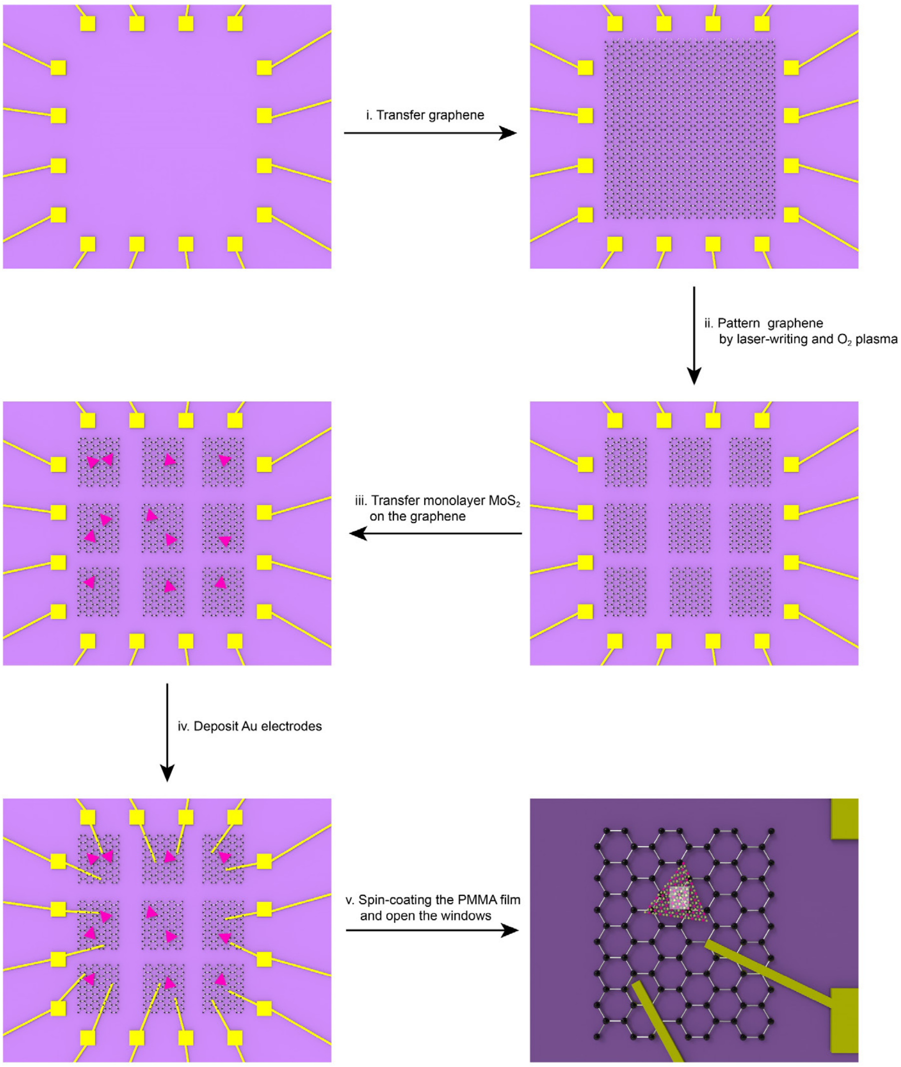

Supplementary Figure 16. Fabrication procedure of the MoS_2/graphene heterostructure microcell. The fabrication process consists of five steps: i) Transfer the high-quality and large-scale CVD-grown graphene monolayer to a SiO_2/Si chip pre-patterned by Au connects; ii) Use laser writing to make the desired pattern on the graphene layer, and then employ oxygen plasm to etch the other parts; iii) Transfer the as-grown monolayer MoS_2 to the obtained graphene pattern through the PMMA-assisted transfer method; iv) Deposit Au/Cr (65/5 nm) electrodes on those graphene patterns near the monolayer MoS_2 using e-beam lithography (EBL) followed by an e-beam evaporation process; v) Spin-coat the PMMA film on the chip and open the desired reaction window on the MoS_2 basal plane through the EBL for the microcell testing.

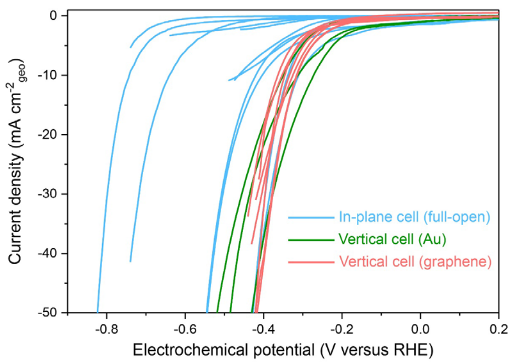

Supplementary Figure 17. Typical polarization curves of three kinds of monolayer MoS_2 on-chip microcells. Blue, green, and red curves represent the HER performance of in-plane cells with full-open windows, vertical cells with Au substrate, and graphene substrate, respectively.

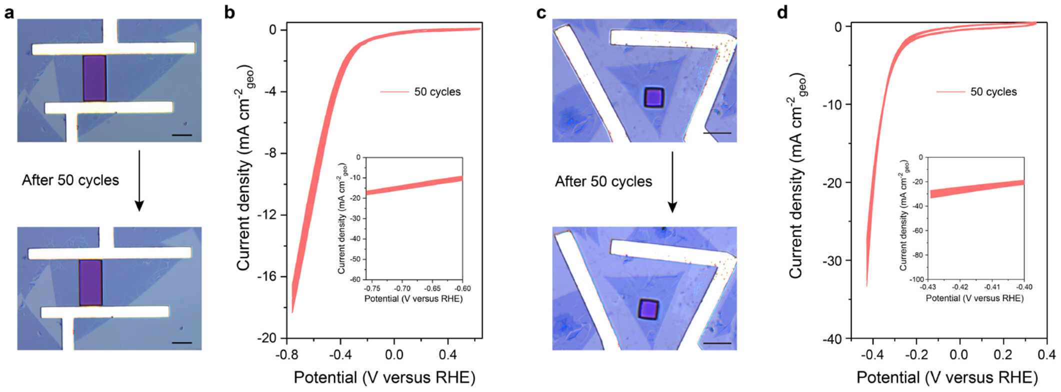

Supplementary Figure 18. Stability of monolayer MoS_2 in full-open and vertical microcells. a and c, The comparison of optical images before and after 50 times cyclic voltammetry in the full-open (a) and vertical (c) microcell. **b and d,** The HER performance stability of full-open (b) and vertical (d) microcell. Inset in (b and d): the zoomed-in view of the corresponding polarization curve. Scale bar: 10 μm.

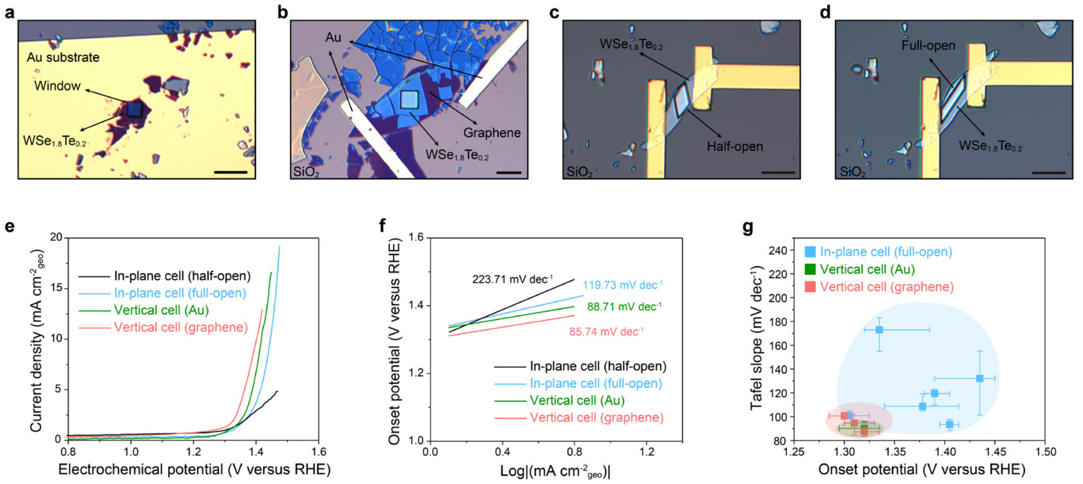

Supplementary Figure 19. Vertical charge transport for $WSe_{1.8}Te_{0.2}$ nanosheet in oxygen evolution reaction. a-d, Optical images of various $WSe_{1.8}Te_{0.2}$ cells, including vertical cells with Au (a) and graphene (b) substrate layers, as well as in-plane cells with half-open (c) and full-open (d) windows. All scale bars are 10 µm. **e-f,** Polarization curves of the current density (e) and the corresponding Tafel slopes (f) for those four kinds of $WSe_{1.8}Te_{0.2}$ cells. **g,** Statistical measurement data of Onset potentials and Tafel slopes obtained from tens of microcells for the OER process. The error bars represent the range of its values in our experiment. Notably, to eliminate the potential effect of $WSe_{1.8}Te_{0.2}$ nanosheet thickness on the performance, we specially selected about 10-nm-thickness nanosheets as the target catalysts (Supplementary Figure 20).

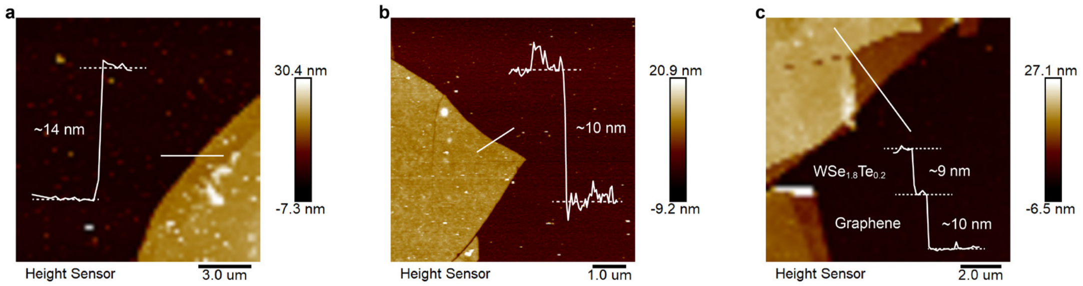

Supplementary Figure 20. AFM images of $WSe_{1.8}Te_{0.2}$ nanosheets in microcells. The thickness of the target catalyst is kept as close as possible to about 10 nm.

Supplementary Note 1: The calculation processes of *in-situ* resistivity of 2D catalysts

The resistivity calculation formula is given by,

$$\rho = \frac{R_{CH}S}{L} = \frac{V_{ds}Wt}{IL} \tag{1}$$

where ρ is the resistivity of the nanosheet, R_{CH} is the channel resistance, S is the cross-sectional area ($= W \times t$, where W is the width and t is the thickness of the nanosheet), V_{ds} is the drain-source voltage, I is the measured current value, and L is the channel length.

Taking the monolayer MoS_2 full-open microcell as the example in Figure 2a, when the electrochemical potential is -0.55 V (vs. RHE), it can be seen that: $V_{ds} = 50\, mV, I = 0.15\, \mu A, W = (W_1 + W_2)/2 = 18\, \mu m, t = 1\, nm, L = 10\, \mu m$. Finally, ρ is calculated to be 0.60 Ω mm.

Supplementary Note 2: The discussion about the threshold between good and bad conductance

The electrocatalytic on-chip microcells have been developed from the top-gated field effect transistors (FETs) and electric double-layer transistors (EDLTs). Both of them contributed to this microcell with well-defined device structures and mature micro-/nano-processing technology, playing a vital role in its emergence and broad applications in electrocatalysis. As a result, the conductance-modulation-based working principle in on-chip microcells should be similar to that in FETs and EDLT, and there exists a threshold, as the reviewer suggested.

Here, we took MoS_2 catalysts in Figure 2a as an example, and extracted the subthreshold slopes (SS) of the full-open and half-open on-chip microcells, as shown in Supplementary Figure 21 below. Note that, $SS = \frac{\partial V_g}{\partial(log_{10} I_{ds})}$, where V_g is the electrochemical potential during the HER and I_{ds} is the conductance current[1]. It can be seen that the full-open one gives an SS of as low as 97 mV dec^{-1}, much better than that of the half-open one (215 mV dec^{-1}), verifying a higher efficiency of the conductance modulation in the former. It is worth mentioning that such an SS is also close to the ideal value in FETs (60 mV dec^{-1}, In the best case, if the efficiency of electrostatic coupling between the gate and the channel region is 100%, then $\frac{C_S}{C_G} = 0$ and $SS = ln(10)\frac{k_B T}{e} = 60\ mV\ dec^{-1}$)), again confirming above conclusions.

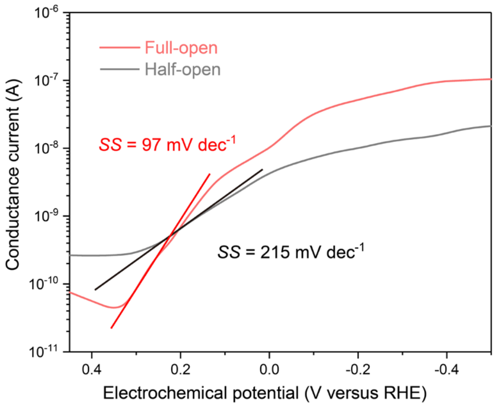

Supplementary Figure 21. Transfer curves and subthreshold slope (*SS*) values of monolayer MoS_2 microcells with the full-open (red curve) and half-open (gray curve) windows. The full-open window shows a much lower *SS* value than the half-open one.

Supplementary Note 3: The penetration depth of surface conductance under EDL-gating

Our previous work suggested that the surface conductance of catalysts can be greatly modulated under the electrical double layer (EDL)[2]. Such a modulation would contribute to two behaviors:

i) High conductivity of the catalyst's surface. Our work shows that EDL enables tuning the conductance by nearly 6 orders of magnitude (Supplementary Figure 22), and a highly-conductive state (0.40 Ω mm) can be obtained, much better than the intrinsic conductivity of TMD materials, e.g., vertical one (2.2-3.3 × 10^4 Ω mm) or in-plane one (9-128 Ω mm).

ii) Penetration depth beneath the semiconductor surface. In theory, such depth is estimated to be about tens of nanometers beneath the semiconductor surface, according to the calculation formula given by[2] $|\psi_1(d)|^2 = \left| A \cdot Ai\left[\left(\frac{2m}{h^2 q^2 \varepsilon^2}\right)^{\frac{1}{3}} \left(q\varepsilon d - \left(\frac{h^2}{2m}\right)^{\frac{1}{3}} \left[\frac{9\pi q \varepsilon}{8}\right]^{\frac{2}{3}}\right)\right]\right|^2$, where $|\psi_1|^2$ is the distribution of density of states, d is the depth beneath semiconductor surface, A is a proportionality constant that can be determined by normalization, m is the mass of electron, q is a unit charge, ε is the electric field strength. In the experiment, we have examined such a penetration depth in the device with a bottom electrode configuration, in line with the above theory results.

Those two behaviors would exclude the possible effect of the interlayer conductivity of multilayer TMD materials on the conductance issue. Furthermore, we strongly recommend that the thickness of 2D catalysts should preferably be less than 20 nm, according to our experiments.

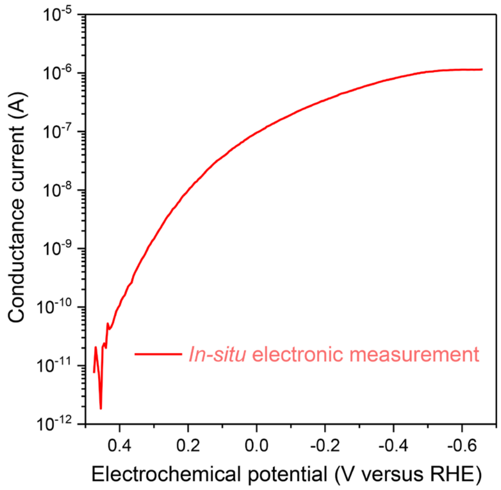

Supplementary Figure 22. The strong modulation ability under EDL-gating in the on-chip microcell. EDL enables tuning the conductance by nearly 6 orders of magnitude.

Supplementary References

1. Ferain, I., Colinge, C.A. & Colinge, J.-P. Multigate transistors as the future of classical metal-oxide-semiconductor field-effect transistors. *Nature* **479**, 310-316 (2011).
2. He, Y., *et al.* Self-gating in semiconductor electrocatalysis. *Nat. Mater.* **18**, 1098-1104 (2019).
